# Blue Native PAGE–Antibody Shift in Conjunction with Mass Spectrometry to Reveal Protein Subcomplexes: Detection of a Cerebellar α1/α6-Subunits Containing γ-Aminobutyric Acid Type A Receptor Subtype

**DOI:** 10.3390/ijms24087632

**Published:** 2023-04-21

**Authors:** Miao Chen, Frank Koopmans, Iryna Paliukhovich, Sophie J. F. van der Spek, Jian Dong, August B. Smit, Ka Wan Li

**Affiliations:** 1 Department of Molecular and Cellular Neurobiology, Center for Neurogenomics and Cognitive Research, Amsterdam Neuroscience, Vrije Universiteit Amsterdam, 1081 HV Amsterdam, The Netherlands; miao.chen@vu.nl (M.C.); frank.koopmans@vu.nl (F.K.); i.paliukhovich@vu.nl (I.P.); sophiejf@hotmail.com (S.J.F.v.d.S.); guus.smit@vu.nl (A.B.S.); 2 Department of Functional Genomics, Center for Neurogenomics and Cognitive Research, Amsterdam Neuroscience, Vrije Universiteit Amsterdam, 1081 HV Amsterdam, The Netherlands; j.dong@vu.nl

**Keywords:** proteomics, protein complex, blue native gel electrophoresis, antibody shift, GABA receptor

## Abstract

The pentameric γ-Aminobutyric acid type A receptors (GABA_A_Rs) are ligand-gated ion channels that mediate the majority of inhibitory neurotransmission in the brain. In the cerebellum, the two main receptor subtypes are the 2α1/2β/γ and 2α6/2β/δ subunits. In the present study, an interaction proteomics workflow was used to reveal additional subtypes that contain both α1 and α6 subunits. Immunoprecipitation of the α6 subunit from mouse brain cerebellar extract co-purified the α1 subunit. In line with this, pre-incubation of the cerebellar extract with anti-α6 antibodies and analysis by blue native gel electrophoresis mass-shifted part of the α1 complexes, indicative of the existence of an α1α6-containing receptor. Subsequent mass spectrometry of the blue native gel showed the α1α6-containing receptor subtype to exist in two main forms, i.e., with or without Neuroligin-2. Immunocytochemistry on a cerebellar granule cell culture revealed co-localization of α6 and α1 in post-synaptic puncta that apposed the presynaptic marker protein Vesicular GABA transporter, indicative of the presence of this synaptic GABA_A_R subtype.

## 1. Introduction

The majority of cellular processes are driven by the (inter-)action of dynamically regulated protein complexes that together form the functional molecular architecture of a cell. The diversity in protein complexes has become apparent in families of ligand-gated ion channels, in which both subunit composition as well as auxiliary proteins contribute to structural and functional diversity [1,2,3]. To identify the constituents of a protein complex, typically a protein complex is immuno-affinity isolated and subsequently characterized by proteomics analysis [4,5]. For in-depth analysis of protein sub-complexes, the protein complexes are often further separated biochemically, for example using blue-native gel electrophoresis (BN-PAGE), and analyzed individually by Western blotting [6,7,8,9,10,11]. In the present study, we explored the GABA_A_ receptor (GABA_A_R) subunit composition in the mouse cerebellum and use BN-PAGE with subunit-specific antibody shifts to distinguish the protein sub-complexes and characterize their constituents with mass spectrometry. 

The GABA_A_R mediates major inhibitory neurotransmission in the mammalian central nervous system. Functional GABA_A_Rs are pentamers that can potentially be assembled from a diverse group of 19 subunits, classified into different subunit classes, namely α1-6, β1-3, γ1-3, δ, ε, π, ρ1-3 and θ subunits [12]. In the cerebellum of the mouse brain, there are two major receptor subtypes; the 2α1/2β/γ2 subtype, which is synaptically localized and mediates phasic inhibition, and the 2α6/2β/δ subtype, which is extrasynaptic and involves in tonic inhibition [13,14,15]. Other subtypes with different combinations of α1/α6 subunits, and either δ or γ2 together with two copies of β subunits, have also been described [16,17]. In contrast, a recent study that examined the assembly rules of GABA_A_Rs reported the segregation of α1 and α6, which ensure the formation of exclusively α1- or α6-containing pentamers. In addition, the δ subunit was found to suppress the α6-containing receptor to assemble with a γ subunit, which further limits the diversity of GABA_A_R subtypes. This observation was supported by an antibody-shift blue native gel electrophoresis study in combination with Western blotting analysis, which revealed the presence of α1- and α6-containing receptors but not an α1α6-containing receptor [7].

In the present study, we focused on the proteome of GABA_A_R α1 and α6 subunits, and the α1 and α6 subunits have 98% and 92% sequence homology between mice and humans, respectively. We showed by classic immunoprecipitation (IP)-based proteomics the existence of GABA_A_R subtypes including one that contains both the α1 and α6 subunit. We then employed an antibody-shift BN-PAGE mass spectrometry (MS) and a novel data analysis pipeline to reveal the sub-complexes of the α6-containing receptor types using antibodies against α6 and γ2. We demonstrated that the majority of α6-containing receptors are composed of α6, β, and δ subunits that lack Neuroligin-2 (Nlgn2). We revealed the presence of α1/α6-containing GABA_A_Rs, the majority of which also lack Nlgn2. Nevertheless, Nlgn2 was present in a minor α6-containing GABA_A_R population that migrated at a higher apparent molecular weight on BN-PAGE. Pre-incubation with γ2 antibody demonstrated predominately the mass shift of α1-containing complexes. Interestingly, a small population of α6-containing complexes at a lower mass range showed mass shift, indicating the feasibility of complex formation containing both α6 and γ2. Lastly, using an immunocytochemical study on a cerebellar granule primary cell culture, we demonstrated the synaptic co-localization of α1 and α6 subunits, which corroborates the biochemical data on the existence of an α1/α6-containing GABA_A_R.

## 2. Results

### 2.1. Identification of the α1 Subunit in α6-Containing GABA_A_R Complexes by Affinity Purification

The classical approach to identifying the protein composition of a complex is to use a specific antibody against the target protein for immuno-precipitation followed by its mass spectrometric analysis. Here, we used one anti-Gabra1 antibody and two different anti-Gabra6 antibodies to specifically reveal the α6-containing receptor composition. Three technical replicates were performed for each IP set. The anti-Gabra6 IPs were repeated once using different batches of animals and analyzed with the same LC-MS setup. Data from the two experiments are shown in Table 1. About 1000 proteins were identified from the IP experiments. As expected, anti-Gabra6 IPs immunoprecipitated a high number of the GABA_A_R subunits α6, β2, β3, and δ, interestingly, α1 and γ2 were also detected, whereas Nlgn2 and Gphn were detected at much lower levels. The replicated experiment demonstrated a similar result, and the complete list of two IP experiments is provided in Tables S1 and S2. To exclude the possibility of cross-reactivity of anti-Gabra6 antibody to other GABA_A_R subunits, we performed anti-Gabra6 IPs on hippocampus lysate. The hippocampus serves as a negative control as it expresses many GABA_A_R subunits including a high abundance of Gabra1 and Gabrb2, whereas Gabra6 is not expressed [18]. Indeed, anti-Gabra6 IPs failed to immunoprecipitate subunits of GABA_A_R from hippocampal extract, indicating the specificity of the antibodies. In Gabra1 IPs from cerebellum extract, the GABA_A_R α1, β2/3, γ2 subunits, and the GABA_A_R-associated proteins Nlgn2 were abundantly pulled down. In addition, the Gabra6 subunit was also detected. Together, these analyses indicate that a subpopulation of cerebellar GABA_A_R contains both Gabra1 and Gabra6, in addition to the two well-described GABA_A_Rs with the composition of 2α1/2β/γ and 2α6/2β/δ.

Of interest, both anti-α6 antibodies immunoprecipitated a complex that contained Nlgn2 and Gabrg2. Gphn was recovered at lower level, and was present in the IPs of hippocampal extract, suggesting that this amount of Gphn was precipitated non-specifically as a background protein. Nlgn2 is a well-described interactor of synaptic GABA_A_R [19,20], but so far it has not been shown to interact with an α6-containing GABA_A_R.

### 2.2. Antibody Shift BN-PAGE-MS Reveals Multiple Forms of α1- and α6-Containing Complexes

A cerebellum extract was divided into two parts and incubated with or without anti-GABA_A_R subunit antibody, respectively. Protein complexes in the extract were subject to BN-PAGE separation according to their masses. After electrophoresis, the gel lane of each sample was cut into 50 slices, proteins in the slice were trypsin digested and analysed by LC-MS with DIA. This acquisition method maximizes protein/peptide completeness with better quantitation across all the gel fractions [21]. Protein relative abundance profiles over the gel fractions were computed from their respective peptide intensity values. We argued that protein components contained in the same complex will co-migrate in the BN-PAGE gel. Therefore, proteins that show the same mass change in their co-migration pattern upon antibody-shift are likely constituents of the same protein complex.

**Figure 1:**
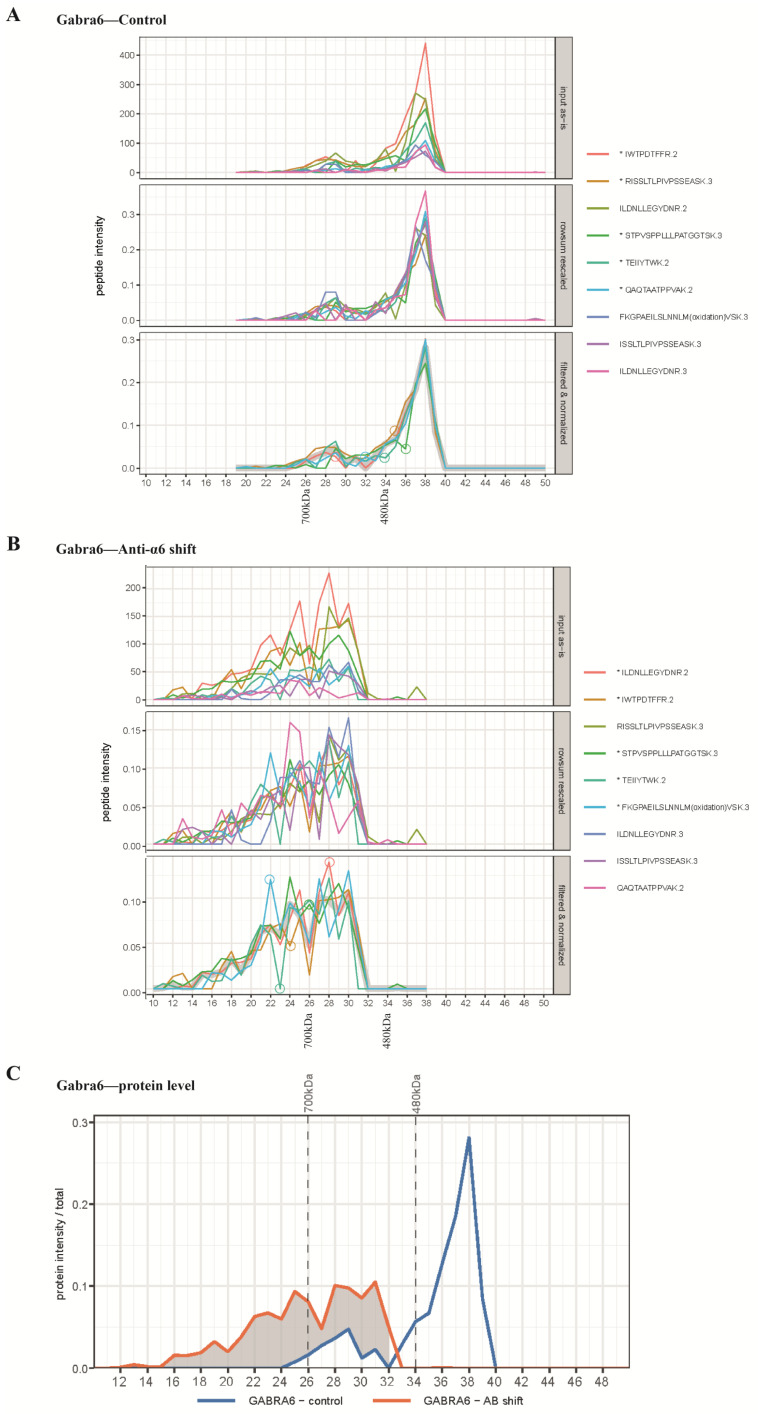
Peptides and protein profiles of Gabra6 in the antibody shift experiment. (**A**) Profiles of identified Gabra6 peptides over the gel slices in the control group, upper panel: top 9 most abundant original peptides profiles (raw data); middle panel: intensity rowsum rescaled peptides profiles, peptide intensity in every gel piece is rescaled by sum of intensity over all gel piece; lower panel: filtered and normalized peptide profiles show the most similar subset of 5 peptides (labelled with an asterisk in the legend) and were selected to represent the consensus profiles. Amino acid sequence and the number of charge states of selected peptides are demonstrated as legends, respectively, and separated by a full stop. (**B**) Profiles of identified Gabra6 peptides over gel slices in the antibody shift group. (**C**) The comparison of protein profiles of Gabra6 between the control group and antibody shift group. Marker proteins were included in each IP-BN-PAGE run as a reference for molecular weight; ferritin light chain 1 peak at the 480 kDa position and ferritin heavy chain 1 at 700 kDa, as depicted on the *x*-axis of the panels in (**A**,**B**) and on top of the graph on panel (**C**).

We next computed the migration pattern of the proteins on the BN-PAGE gel based on the intensities of the tryptic peptides across all the gel slices. Different peptides derived from a protein are detected in the MS with different intensities, depending on their physico-chemical properties, with the lower intensity peptides expected to have higher noise than the high intensity ones. We therefore interrogated all the peptides from a protein (Figure 1A,B, upper panel) and arrived at a consensus profile for all gel fractions using a 3-step process: (1) rescale peptide intensities such that Euclidean distances between peptides are minimized (Figure 1A,B, middle panel), (2) find the subset of N peptides by an unsupervised algorithm (depending on available peptides) that has the most similar consensus profile to the gel fractions (Figure 1A,B, bottom panel), (3) compute a protein-level consensus profile by fitting a Loess trendline with narrow span (Figure 1C). The peptides of interested selected by our unsupervised algorithm for each protein and experiment are provide in Table S3.

For the calibration of the migration pattern on the BN-PAGE, we used the spiked-in marker proteins, the ferritin heavy and light chain 1 that migrated at 700 kDa and 480 kDa, respectively. The majority of the native α6-containing complexes migrated at ~450 kDa, and about 10% at ~680 kDa (Figure 1C), as estimated by extrapolation by eye. Pre-incubation with the anti-α6 antibody caused a complete shift of the 450 kDa sub-complex to higher mass at >700 kDa, whereas the minor 680 kDa sub-complex was shifted toward a higher molecular weight position. 

**Figure 2:**
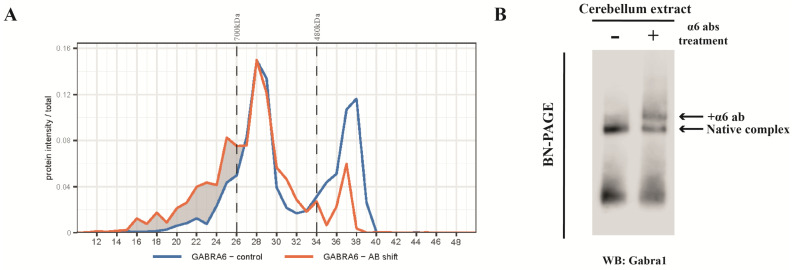
Migration of GABA_A_R α1 subunit after anti-α6 antibody shift identified by mass spectrometry and immunoblotting. (**A**) The comparison of protein profiles of the α1 subunit between control and anti-α6 subunit antibody shift groups. Markers for 480 and 700 kDa were indicated on the top of panel and gel slice number is presented on the *x*-axis of the panels. (**B**) BN-PAGE of anti-Gabra6 antibody shifted cerebellum lysate and control lysate were transferred on PVDF membrane and immunostained for Gabra1. The α1 subunit was identified in the control group and antibody shifted group as indicated.

As detected in the IP experiment (Table 1), the Gabra1 subunit was present in the α6-IP. To specifically confirm that the α1 and α6 subunits can be assembled into the same receptor, we treated cerebellum extract with anti-α6 antibodies prior to BN-PAGE. Figure 2A shows that the α6 antibody causes a shift of a sub-population of α1-containing complexes at mainly 450 kDa, and, to a smaller extent, at the higher masses. This implies that at 450 kDa there is a considerable amount of the α1/α6-containing sub-complex. The presence of the α1/α6-containing sub-complex was validated independently by immuno -blotting analysis (Figure 2B). 

It should be noted that it is difficult to directly relate the increase in mass of the antibody-bound protein complex to the extent of retarded migration on the BN gel because migration in the BN gel depends not only on the total mass of the complex but also on its structural property. Furthermore, there seem to be multiple peaks in the antibody shift sample. This may be underlay by the fact that there are several subtypes of the GABA_A_R complexes, which may contain (1) two α1, (2) two α6, or (3) one α1 and one α6, which may or may not be associated with Nlgn2. The binding of the antibody to the different complexes caused mass shift, which may overlap, in part, with other sub-complexes. The heterogeneity of the mass shift might further be underlay by interaction with one or two antibody molecules. Together, the protein masses of the complexes after antibody shift are generally less well defined.

### 2.3. BN-PAGE-MS Antibody Shift to Reveal the Migration Patterns of Other GABA_A_R Subunits and Their Associated Proteins

**Figure 3:**
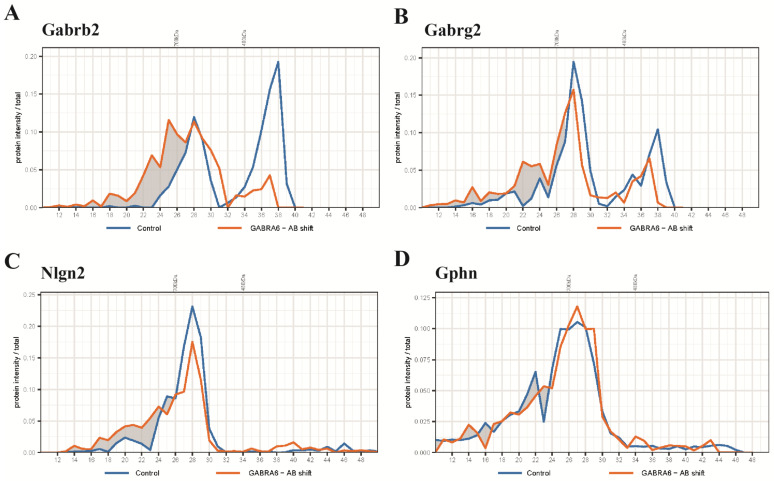
Protein profiles of selected proteins from an anti-α6 antibody shift experiment identified by mass spectrometry. (**A**) Pre-incubation of cerebellar extract with anti-α6 antibody shifted β2-containing complexes globally to a higher molecular weight in the BN. (**B**) The antibody shift reduced the amount of γ2-containing complex at 450 kDa and caused a small fraction of the complex at 680 kDa to increase in weight to around 750 kDa. (**C**) Nlgn2 demonstrated an up-shift from 680 kDa to 750 kDa and the shifted fraction overlapped with the shifted γ2-containing complex. (**D**) This antibody shift did not affect the migration pattern of Gphn.

To further identify proteins potentially residing in the α1/α6-containing sub-complex, we interrogated migration profiles of other proteins of interest after anti-α6 antibody shift. First, we observed that a high proportion of Gabrb2 shifted (Figure 3A), which is in agreement with the presence of this subunit in the GABA_A_R (Table 1). Gabrg2 is the main component of the α1-containing complex, which is considered to be excluded from the α6 containing receptor [7]. Here, a small population of this subunit showed a molecular weight shift (Figure 3B), suggesting that some of the α6-containing GABA_A_R contains Gabrg2. Nlgn2 and Gphn are marker proteins of the synaptic inhibitory synapse. The main population of the α6-containing subcomplex resided at 450 kDa; at this size class, the complex did not co-migrate with Nlgn2, nor Gphn. Nlgn2 was present predominantly in the complex that migrated around 680 kDa, but not 450 kDa (Figure 3C). Gphn was not affected by antibody shift (Figure 3D). This is in agreement with the IP experiment showing no co-immunoprecipitation of Gphn with the anti-α6 antibody (Table 1). Taken together, the majority of the α6-containing sub-complex, which migrated at 450 kDa, was the naked form of the GABA_A_R without associated proteins. 

After anti-α6 antibody incubation, a small part of α6-containing GABA_A_Rs migrated at >700 kDa, which was possibly associated with Nlgn2 (Figure 3C). Considering that only a small part of the α6-containing receptor migrated as high-MW complexes, these data confirm the previously identified low amounts of Nlgn2 in α6 Ips using anti-Gabra6b antibody (Table 1).

**Figure 4:**
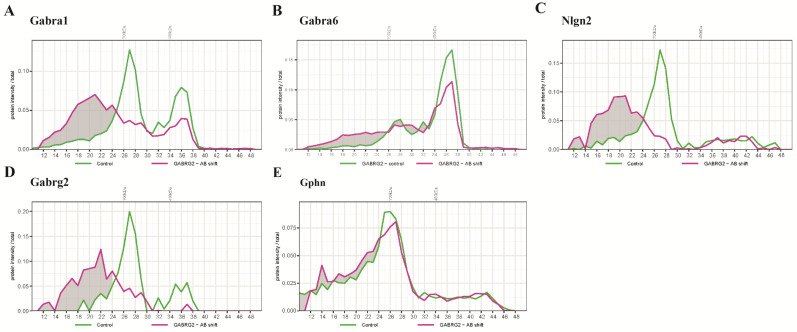
Protein profiles of selected proteins from an anti- γ2 antibody shift experiment identified by mass spectrometry. (**A**) Pre-incubation of cerebellar extract with anti-γ2 antibody shifted large proportion of α1-containing complexes to higher masses in the BN. (**B**) The antibody shift slightly reduced the amount of an α6-containing complex at 450 kDa, and caused the increase in the amount of a complex > 730 kDa. (**C**) The migration of a γ2-containing complex at 680 kDa was accompanied by a shift of Nlgn2 toward higher masses. (**D**) Nearly all of the γ2 subunits were up-shifted toward higher mass. (**E**) The anti-γ2 antibody shift did not affect the migration pattern of Gphn.

Both 2α1/2β2/γ2 and 2α6/2β2/δ subtypes contribute to the intensities of the β2 subunit across the BN fractions. The γ2 subunit is mainly associated with the 2α1/2β/γ2 form [12]. To examine whether it may also be present in the α6-containing receptor isoform, we performed an antibody-shift BN-PAGE-MS using an anti-γ2 antibody (Figure 4). Indeed, the intensities of the α1 subunit at 680 kDa migrated to >730 kDa (Figure 4A). A substantial amount of Gabra6 remained unshifted, indicating that a large population of sub-complexes did not contain γ2, represented by the highly abundant 2α6/2β/δ complex. Interestingly, a small population of α_6_ migrated to >730 kDa, suggesting that a population of GABA_A_R at 680 kDa comprised α6 and γ subunits. The concomitant appearance of Nlgn2 at higher masses is in agreement with the antibody-shifted α1 and γ2 subunits (Figure 4C). Surprisingly, this antibody did not affect the migration pattern of Gphn (Figure 4E), suggesting a weak, or absence of, direct interaction [22,23]. 

### 2.4. The Subcellular Localization of the α1- and α6-Subunit-Containing Receptors in Cultured Cerebellar Neurons

Whereas the localization of the synaptic 2α1/2β/γ2 and extrasynaptic 2α6/2β/δ receptors is well documented, the localization of the α1/α6-containing complex is less studied. We therefore examined the spatial distribution of α1 and α6 along dendrites and synaptic sites using immunocytochemistry on a cerebellar granule culture. 

**Figure 5:**
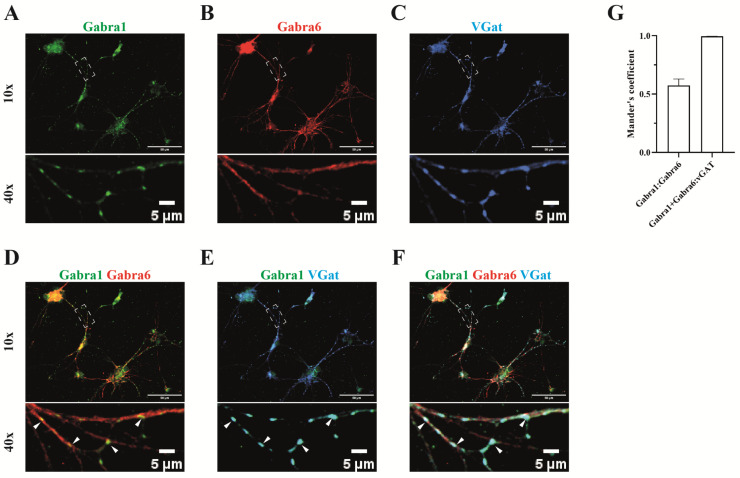
Synaptic localization of α1/α6 containing GABA_A_ receptor. The distribution of both α1 and α6 subunits was investigated on the cerebellar granule cells (**A**,**B**) and both subunits were each co-stained with the inhibitory GABAergic presynaptic neuronal marker protein vGAT (**C**). α6 immunoactivity was present along the neurite. α1 was predominately present in synapses opposing the pre-synaptic vGAT (**E**), where α6 also often co-localized (indicated by arrow heads, **D**,**F**). (**G**) Manders’ coefficient indicates the overlap of α1 subunit (green channel) with α6 subunit signal (red channel). The co-localized puncta between the α1 and α6 subunits over vGat (blue channel) are indicated by Manders’ coefficients as w/vGat.

Consistent with the known synaptic localization of α1, the α1 immunoreactivity was present in puncta that were always opposing the presynaptic marker protein vGat (SLC32A1) (Figure 5A,C,E), suggesting a postsynaptic localization. The widespread distribution of α6 along the neurites is in agreement with the extra-synaptic localization of the 2α6/2β/δ (Figure 5B). Overlap of α1 and α6 immunoreactivity was observed in many puncta (Figure 5D,F,G), supporting our data obtained on α1/α6-containing receptors as identified by proteomics, and supporting the view that the α1/α6-containing receptors are synaptically localized.

## 3. Discussion

In this study, we combined antibody shift assay and BN-PAGE to specifically inspect the migration profiles of key proteins of GABA_A_R complexes and confirmed the presence of the GABA_A_R subtype containing both α1 and α6 subunits. In addition, a small population of α6-containing GABA_A_R appear to assemble with the γ2 subunit. The typical synaptic Nlgn2 is also shown to interact with α6-containing GABA_A_R. Using immunocytochemical staining we demonstrated that α1 and α6 co-localize at the synapse.

To identify the composition of a protein complex, affinity purification-based proteomics has been extensively used [4,5,24,25,26,27]. Typically, specific antibodies against the protein of interest are used for IP from tissue extract, in which the co-IPed proteins are subsequently identified by mass spectrometry. However, most proteins are present in multiple complexes with specific cellular and sub-cellular spatiotemporal distribution patterns [28,29]. A typical AP approach would not distinguish the sub-complexes; rather, it would report an average protein composition recovered simultaneously from all the immunoprecipitated sub-complexes. Therefore, an alternative approach, as used here, is to employ BN-PAGE to separate intact protein complexes according to size, i.e., proteins embedded in the same complex will co-migrate on the gel [30], whereas protein constituents are then identified by MS. This approach has been successfully applied on organelles, such as mitochondria, to reveal distinct protein complexes [6,31], but may not work well in tissue extract that contains a multitude of proteins with incidental co-migration of proteins from different complexes but with similar size. To address this limitation, we recently reported the combination of IP followed by BN-PAGE-MS to achieve a more specific analysis of targeted protein complexes [4,32]. This IP step can isolate a protein of interest and allow for a more precise identification of protein co-migration on the BN-PAGE as it reduces complexity and enriches for the target protein complex. A caveat is that the affinity isolated protein complex needs to be released from the antibody before it can be run on the BN-PAGE, which critically depends on the binding affinity between the antibody and the target protein. On one hand, high affinity interaction is an advantage for the capture of the target protein and the associated proteins from the extract; on the other, it may hinder the elution of the protein complex from the antibody that is necessary for loading onto the BN-PAGE and the subsequent MS analysis. Theoretically, this limitation can be alleviated by antibody-shift in combination with BN-PAGE mass spectrometry. Here, the extract is incubated with or without the antibody of interest and run on BN-PAGE. Proteins from the target complex would be shifted to higher mass due to the added mass of the antibody, which is indicative of taking part in the same complex, which can be detected and quantified by mass spectrometry.

In the mouse cerebellum, the most abundant GABA_A_R subunits are the synaptic 2α1/2β/γ2 and the extra-synaptic 2α6/2β/δ [16,33]. As previously reported, the α1 and α6 subunits, as well as the γ2 and δ subunits, are mutually exclusive pairs in a GABA_A_R [34]. Nevertheless, previous IP-based experiments indicated the potential existence of α1α6-containing GABA_A_Rs [16,35]. Our IP experiments are consistent with these previous studies. Importantly, we further validated these findings independently by an antibody shift BN-PAGE-MS approach and demonstrated the existence of multiple GABA_A_R isoforms with a large proportion of naked 2α_6/_2β/δ composition without any associated proteins, and a smaller amount associated with Nlgn2, whereas the 2α_1/_2β/γ2 is mainly associated with Nlgn2. We detected the α1/α6-containing receptor and revealed the co-localization of α1 and α6 subunits in the synapses of cerebellar granule cells in culture by immunocytochemical staining. We therefore argue that α1 and α6 subunits are not mutually exclusive and together can be present in a GABA_A_R receptor, albeit at a lower abundance than the 2α1/2β/γ2 and the 2α6/2β/δ types.

The physiological and pharmacological properties of the GABA_A_Rs are critically dependent on the receptors’ subunit composition [36], as demonstrated by the tonic inhibition elicited by the extra-synaptic 2α6/2β/δ receptor and the phasic 2α1/2β/γ2 synaptic receptor. GABA_A_R subtypes were shown to be pharmacologically distinct and modulated differentially by a variety of drugs [12]. For example, the 2α1/2β/γ2 receptor demonstrates a strong sensitivity to benzodiazepine, whereas the 2α6/2β/δ receptor does not [37,38]. As such, the GABA_A_ receptor is the target of many drugs of medical importance, being used as anticonvulsants, depressants, anxiolytics, and sedative–hypnotics [39]. Future experiments are needed to define the physio-chemical and the biological properties of these novel α1/α6-containing GABA_A_R subtypes.

Changes in GABA_A_R synthesis, delivery, and anchoring at the membrane may have a significant effect on normal brain function and these biological processes are closely regulated by receptor-associated proteins [40,41]. In the current study, we further examined the two major GABA_A_R interacting proteins, Nlgn2 and Gphn. Nlgn2 is a synapse-specific adhesion protein that is known to interact with synaptic GABA_A_R subtypes and receptor scaffolds [42,43]; dysfunction of this protein has been implicated in autism [42]. As expected, 2α1_/_2β/γ2 is mainly associated with Nlgn2. However, Nlgn2 was also immunoprecipitated from anti-α6 IPs and was found to shift with both α6 and γ2 in BN-PAGE, albeit for a small fraction of the α6-containing GABA_A_R population. We then demonstrated the co-localization of α6 and α1 with the presynaptic protein VGat, which suggests that Nlgn2 and the α1/α6 receptor subtype interacts at the synapse. Gphn is known to play a crucial role in the formation of GABAergic synapses and involves the post-synaptic accumulation of GABA_A_R [44,45]. However, Gphn failed to be detected in the IP experiments, nor was it shown to shift in the antibody-shift BN-PAGE experiments. It has been reported that Gphn interacts weakly with GABRA_A_R or is present in a complex distinct from GABA_A_R–Nlgn2 [22,46]. This is in sharp contrast to the strong interaction of Gphn to another type of inhibitory receptor, the glycine receptor [4,47].

## 4. Materials and Methods

Brain material: All experiments were approved by the Animal Users Care Committee of the Vrije Universiteit Amsterdam and were performed in accordance with the relevant guidelines and regulations.

Immunoprecipitation/SDS-PAGE Fractionation: Mouse cerebellum was homogenized using a potter and pestle (Sartorius, Göttingen, Germany; 12 strokes, 900 rpm) in 1% maltose–neopentyl glycol (MNG) buffer, containing 25 mm HEPES, 150 mm NaCl (pH 7.4), and proteinase inhibitor (Roche, Basel, Swiss), and incubated for 1 h at 4 °C. After centrifugation at 20,000× *g*, 10 μg antibody was added to the supernatant and incubated overnight at 4 °C. The antibody was captured by 40 μL protein A/G plus agarose beads (Santa Cruz, Dallas, TX, USA). After washing four times with 0.1% MNG containing 25 mm HEPES and 150 mm NaCl (pH 7.4), the beads were mixed with SDS sample buffer and heated to 98 °C for 5 min. Proteins were separated on a 10% SDS polyacrylamide gel and stained with Coomassie Blue. Each sample lane was cut into three fractions, and each fraction was further cut into small gel pieces and transferred to a 96-well plate (0.45 μm filter; MultiScreen-HV 96-well filter plate from Millipore, Burlington, MA, USA). Gel pieces were destained by sequential incubation with 100 μL 50 mM NH_3_HCO_3_/50% acetonitrile and then 75 μL 100% acetonitrile. For each step, brief centrifugation of the plate was performed to remove the supernatant. Gel pieces were subsequently incubated with MS grade endolysC/trypsin (Promega, Madison, WI, USA) at 37 °C overnight. One hundred microliters of 0.1% TFA in 50% acetonitrile was added to each fraction, incubated for 40 min, collected in an Eppendorf tube, and dried in the SpeedVac. 

The commercial antibody used was from NeuroMab, Davis, CA, USA, GABA_A_ α1 antibody (N95/35, AB_106697873) and GABA_A_ α6 antibody (N229A/32, AB_2491091, labeled as Gabra6-a). We obtained a custom-made antibody from Genscript (Piscataway, NJ, USA) against Gabra6 (peptide sequence: CSQKAERQAQTAATPPVAKSKASE; labeled as Gabra6-b).

Blue native-PAGE (BN-PAGE) and antibody shift: BN-PAGE was performed as described previously [48]. In brief, mouse cerebellum was solubilized with 1% MNG buffer as described above. Ten micrograms of solubilized protein was mixed with 5 μL 4× BN loading buffer, 0.5 μL of molecular weight marker (Thermo Fisher, Waltham, MA, USA), and 1 μL 5% G-250 Coomassie blue and loaded on a pre-cast Invitrogen NativePAGE 4–16% Bis–Tris Gel (Thermo Fisher, Waltham, MA, USA). The gel was run at 4 °C and 150 V for 1.5 h, followed by 250 V for 1 h. 

For the antibody shift, 10 μg cerebellum homogenate was incubated with 4 μg antibodies for 1 h before it was loaded on the BN gel. Gabra6-a antibody and Gabrg2 antibody (Neuromab, Davis, CA, USA, N452/69, AB_2617122) were used. Gel running conditions were the same as above. 

After running, the gel was fixed overnight in 200 mL of 50% ethanol, 7% acetic acid, and 3% phosphoric acid. On the second day, the gel was washed three times in water and cut with a grid cutter (The Gel Company, San Francisco, CA, USA) into 70 equally sized pieces. Each piece was transferred individually to a well in a 96-well filter plate (0.45 μm filter; MultiScreen-HV 96-well filter plate from Millipore, Burlington, MA, USA).

The gel pieces were treated with 100 μL reducing agent Tris(2-carboxyethyl) phosphine hydrochloride at 37 °C for 30 min, followed by incubation with 100 μL of a cysteine blocker, methyl methanethiosulfonate, at RT for 15 min, and finally washed with 100 μL 50 mM NH_3_HCO_3_ at RT. For each step, brief centrifugation of the plate was performed to remove the supernatant. The subsequent protein destaining, digestion, and peptide harvest processes were performed as described previously.

For immunoblotting, native cerebellum homogenate and homogenate treated with antibodies were run on the same gel at 4 °C, 150 V for 1.5 h, followed by 250 V for 1 h. Protein complexes on the gel were transferred onto a PVDF membrane overnight at 4 °C, 40 V. The membrane was fixed using 8% acetic acid and blocked with 5% non-fat milk in Tris-buffered saline (pH 7.4) with 0.1% Tween-20 (TBS-T), then the membrane was incubated with primary antibody against Gabra1 (Milipore, Burlington, MA, USA, AB5592, 1:1000) at 4 °C, overnight. After three washes with TBS-T buffer, the blot was incubated with HRP-conjugated Goat anti-Rabbit IgG secondary antibody (Dako, Santa, Clara, CA, USA, 1:1000) in 3% non-fat milk added to TBS-T buffer. The blot was washed three times, incubated with SuperSignal West Femto Chemiluminescent Substrate (Thermo Fisher, Waltham, MA, USA), and scanned on an Odyssey Fc scanner (Licor Biosciences, Lincoln, NE, USA)

HPLC-ESI MS/MS: Two LC-MS setups were used. The sample analysis using an Ultimate 3000 LC system (Dionex, Thermo Scientific, Waltham, MA, USA) coupled to a TripleTof 5600^+^ Mass spectrometer (Sciex, Framinghan, MA, USA) was performed as previously described [4,49,50]. Using this setup, immunoprecipitation samples and the Gabra6 antibody shift and its control group were analyzed. The former was analyzed in data-dependent mode and the latter was analyzed in data-independent mode. In brief, the samples were fractionated in a capillary LC column with a linearly increased gradient of acetonitrile from 5% to 30% in 35 min, to 40% at 37 min, and to 90% for 10 min, at a flow rate of 5 μL/min. The eluted peptides were electro-sprayed into a TripleTof 5600^+^ Mass spectrometer (Sciex, Framinghan, MA, USA) at 5500 V, ion source gas at 2 psi, curtain gas at 35 psi, and an interface heater temperature of 150 °C. 

Evosep One coupled to a TimsTOF Pro 2 mass spectrometer was used for the analysis of the anti-Gabrg2 antibody shift BN-PAGE samples. In brief, the peptide solution was transferred to an Evotip and run on a 15 cm × 75 µm, 1.9 µm Performance Column (EV1112 from EvoSep, Odense, Danmark) using the Evosep One liquid chromatography system with the 30 samples per day program. Peptides were electro-sprayed into the TimsTOF Pro 2 mass spectrometer (Bruker, Billerica, MA, USA) and analyzed with diaPASEF [51]. The MS scan was between 100 and 1700 *m*/*z*. The Tims settings were 1/Ko from start to end and between 0.6 and 1.6 V.s/cm^2^, ramp time 100 ms, accumulate time 100 ms, and ramp rate 9.42 Hz.

Data analysis: Raw DDA data were searched against the UniProt Mouse proteome (release 2018-04) using MaxQuant (1.6.3.4) [52] in default settings with iBAQ enabled. Raw diaPASEF data were searched against a virtual spectral library generated from the UniProt Mouse proteome (release 2018-04) using DIA-NN 1.8 [53]. Protein inference was set to isoform and cross-run normalization was activated. The precursor charge range was 2–4. A fixed modification of UniMod: 39,45.987721,C was used, which represents an MMTS modification of the cysteine residue.

Immunocytochemistry: Cerebella from P6 wildtype mice (C57Bl6 mice, Vrije Universiteit Amsterdam) were dissected and incubated in Hank’s balanced salts solution (HBSS) (1M, Thermo Fisher, Waltham, MA, USA) and digested in buffer containing 7 mM HEPES, pH 7.4, and 0.25% trypsin (Thermo Fisher, Waltham, MA, USA). After the cerebellum was washed three times in HEPES buffer and twice in neurobasal medium (Thermo Fisher, Waltham, MA, USA), neurons were triturated with Pasteur pipettes, and neuronal concentration was calculated in a Fuchs-Rosenthal chamber. Additionally, 12.5 K/well cells were plated for culturing in a neurobasal medium supplemented with 2% B-27 (Thermo Fisher, Waltham, MA, USA), 2% HEPES solution, 0.25% glutamine (200 mM, Thermo Fisher, Waltham, MA, USA), and 1% penicillin/streptomycin on a 96-well glass plate bottom, which was coated in poly-d-lysine/laminin (Sigma-Aldrich, St. Louis, MO, USA) and incubated at 37 °C and 5% CO_2_.

Neurons at DIV7 were fixed using 4% paraformaldehyde and 1% sucrose in phosphate-buffered saline (PBS) and pH 7.4 (Thermo Fisher, Waltham, MA, USA) for 20 min and washed twice with PBS. Cells were permeabilized with 0.5% Triton-X (Sigma-Aldrich, USA) for 20 min on a shaker at RT and blocked with 2% goat serum and 0.1% Triton-X in PBS for 1 h at RT. For primary antibody incubation, all antibodies were diluted to 1:1000 in blocking solution and were used to incubate with the cells at 4 °C overnight. Cells were washed three times with PBS and immediately incubated with a secondary antibody in a blocking solution with the ratio of 1:400 (*v*/*v*) at RT for 2 h in dark. After washing three times in PBS, cells were ready for immunocytochemistry analysis.

For confocal imaging, the following antibodies were used: anti-Gabra6 b (Genscript, Piscataway, NJ, USA, 1:1000), anti-Gabra1 (Neuromab, Davis, CA, USA, 1:1000), and anti-vGAT (SySy, Göttingen, Germany, 131-004, 1:1000). Cerebellar granule neurons were incubated with primary antibodies overnight at 4 °C. Cells were then washed with PBS and incubated with corresponding Alexa conjugated secondary antibodies (1:400) with a thin foil cover for 1 h at RT. Images were captured using a Nikon Eclipse Ti microscope confocal laser scanning microscope (40× objective; NA 1.3) with NIS-Elements 4.30 software. Quantification of colocalization was performed by ImageJ for *Pearson correlation* coefficient and Mander’s coefficient analysis via the JACop plugin [54].

## Figures and Tables

**Table 1:** Anti-GABA_A_R α6 and α1 subunits IPs. Immunoprecipitation data were presented as ‘average iBAQ value and standard deviation|averaged iBAQ values as % of the bait protein’. NA, not available. (*n* = 3 technical replicates per antibody).

Gene Names	Batch 1				Batch 2		
	IPs on Cerebellum Lysate			IPs on Hippocampal Lysate	IPs on Cerebellum Lysate		IPs on Hippocampal Lysate
	Gabra1	Gabra6a	Gabra6b	Gabra6b	Gabra6a	Gabra6b	Gabra6b
Gabra1	180,790 ± 20,398|100%	2487 ± 385|8.9%	18,008 ± 4424|19.1%	6 ± 12|NA	6248 ± 1285|6.7%	11,343 ± 2100|14.4%	0|NA
Gabra6	56,914 ± 8010|31.5%	27,938 ± 1797|100%	94,089 ± 18,976|100%	0 ± 0|NA	92,982 ± 15,344|100%	79,072 ± 12,676|100%	0|NA
Gabrb2	255,366 ± 20,281|141.3%	25,306 ± 2234|90.6%	79,766 ± 13,765|84.8%	0 ± 0|NA	76,551 ± 15,043|82.2%	88,527 ± 21,864|111.2%	0|NA
Gabrb3	37,074 ± 4005|20.5%	4748 ± 602|17%	19,323 ± 5003|20.5%	0 ± 0|NA	21,835 ± 4511|23.4%	18,904 ± 4756|23.7%	0|NA
Gabrd	31,971 ± 3319|17.7%	9724 ± 1041|34.8%	35,276 ± 5229|37.5%	0 ± 0|NA	39,517 ± 4543|43.4%	47,333 ± 1218|60.8%	0|NA
Gabrg2	81,516 ± 13,238|45.1%	1487 ± 492|5.3%	7641 ± 831|8.1%	0 ± 0|NA	7621 ± 456|8.3%	8403 ± 297|10.8%	0|NA
Gphn	171 ± 51|0.1%	7 ± 9|0%	0 ± 0|0%	4 ± 8|NA	113 ± 33|0.1%	9 ± 16|0%	7 ± 1|NA
Nlgn2	58,868 ± 8423|32.6%	695 ± 132|2.5%	4571 ± 934|4.9%	0 ± 0|NA	234 ± 119|0.3%	87 ± 79|0.1%	0|NA

## Data Availability

Not applicable.

## Supplementary Materials

The following supporting information can be downloaded at: https://www.mdpi.com/article/10.3390/ijms24087632/s1.
